# Supplementary figures and images for: Effects of Bradyrhizobium Co-Inoculated with Bacillus and Paenibacillus on the Structure and Functional Genes of Soybean Rhizobacteria Community
Source: Genes (Basel). 2022 Oct 22;13(11):1922. doi: 10.3390/genes13111922 (PMC9689485; doi:10.3390/genes13111922)

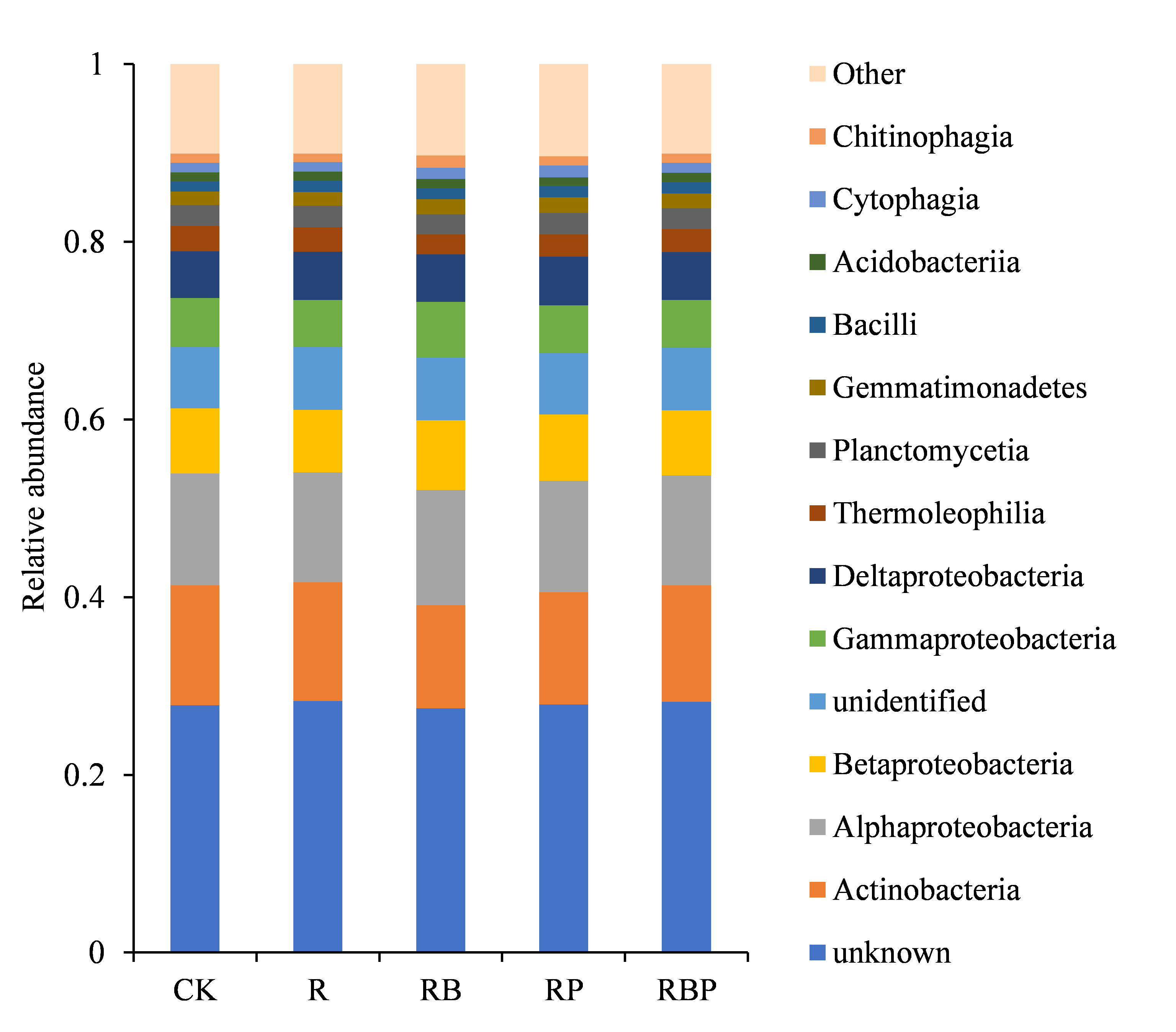

Supplement: Supplementary file 1 [file genes-13-01922-s001.zip › Figure S1. Relative abundance of predominant bacteria at class level.tif]

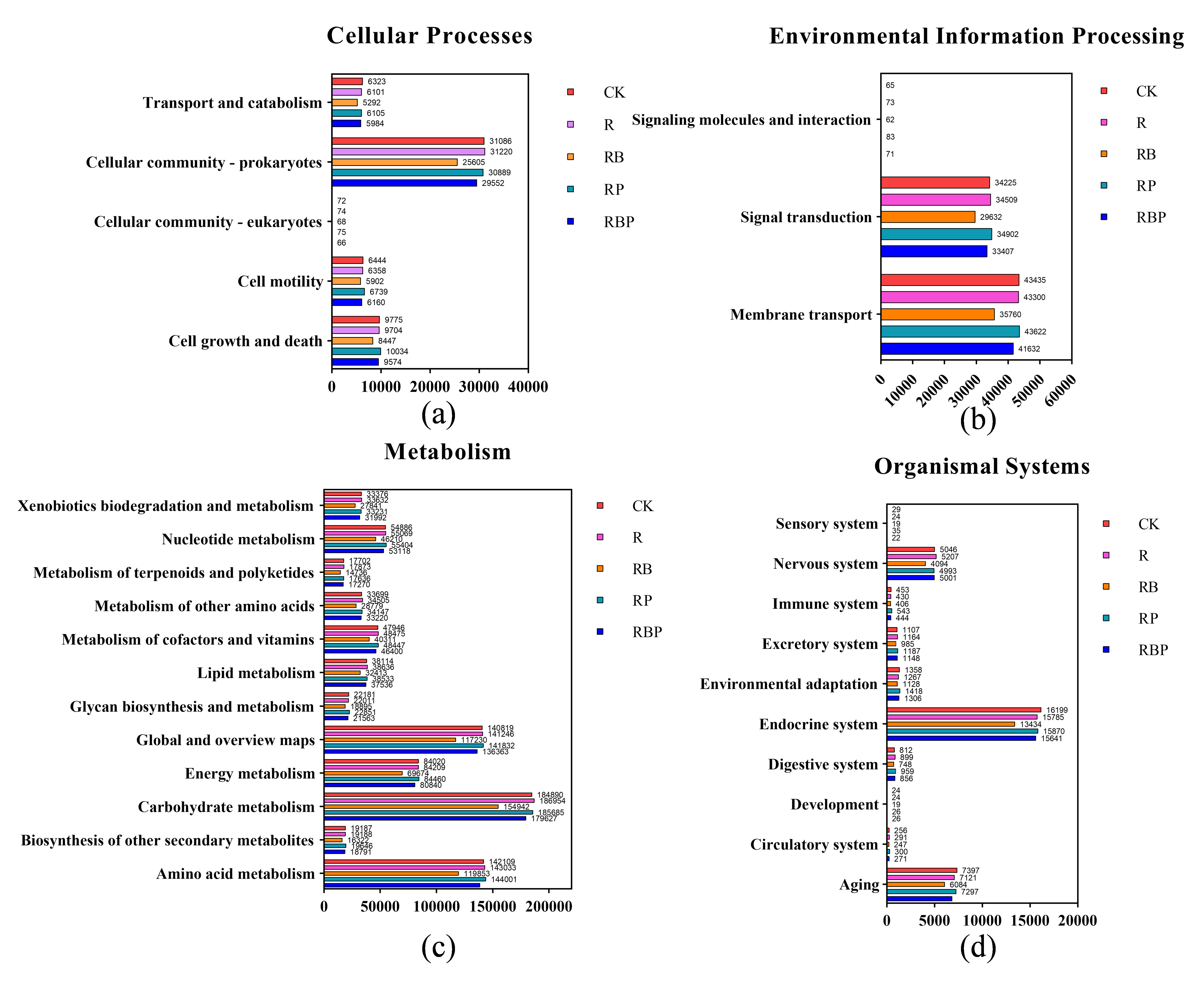

Supplement: Supplementary file 1 [file genes-13-01922-s001.zip › Figure S2. Classification results of gene function based on KEGG database.tif]
